# Supplementary material for: Are Escherichia coli causing recurrent cystitis just ordinary Uropathogenic E. coli (UPEC) strains?
Source: bioRxiv. 2023 Nov 9:2023.11.08.566351. Preprint. [Version 1] doi: 10.1101/2023.11.08.566351 (PMC10659292; doi:10.1101/2023.11.08.566351)
Supplement: 1 [file NIHPP2023.11.08.566351v1-supplement-1.pdf]

bioRxiv preprint doi: https://doi.org/10.1101/2023.11.08.566351; this version posted November 9, 2023. The copyright holder for this preprint (which was not certified by peer review) is the author/funder, who has granted bioRxiv a license to display the preprint in perpetuity. It is made available under aCC-BY 4.0 International license.

Supporting informations

Fig S1. Distribution of allelic variants of 23 virulence factor determinants in RCI and SCI groups. Each color represents an allelic variant of the corresponding gene.

Fig S2. Comparison of iRCI (red) and last rRCI (orange) doubling times in two media: lysogenic broth (LB) and artificial urinary medium (AUM). Red asterisks represent outliers. Black asterisk represents significant median doubling time differences between iRCI and rRCI ($p < 0.05$).

Fig S3. Comparison of iRCI (red) and last rRCI (orange) biofilm formation in two media: lysogenic broth (LB) and artificial urinary medium (AUM). Red asterisks represent outliers. Black asterisk represents significant median biofilm formation differences between iRCI and rRCI ($p < 0.05$).

Fig S4. Comparison of invasion rate of bladder epithelial cells by iRCI (red) and last rRCI (orange) in a given relapse series. Names of relapse series are indicated above each boxplot. Red asterisks represent outliers. Black asterisks represent significant median biofilm formation differences between iRCI and rRCI ($p < 0.05$).

Table S1. Sequencing, typing and pairing data for 85 isolates included in the study

Table S2. List and gene annotation of the 7 lost plasmids in 5 RCI series

bioRxiv preprint doi: https://doi.org/10.1101/2023.11.08.566351; this version posted November 9, 2023. The copyright holder for this preprint (which was not certified by peer review) is the author/funder, who has granted bioRxiv a license to display the preprint in perpetuity. It is made available under aCC-BY 4.0 International license.

Table S3. List and functional annotation of the 13 overtime conserved SNPs identified in 6 isolates from 6 RCI series

Table S4. Composition of AUM adapted from Brooks and Keevil (1997)
